# Speech timing metrics as cross‐linguistic markers of Alzheimer's and cognitive decline: Evidence from Kiswahili, Spanish, and Portuguese speakers

**DOI:** 10.1002/alz70856_106105

**Published:** 2026-01-07

**Authors:** Adolfo M Garcia

**Affiliations:** ^1^ Cognitive Neuroscience Center (CNC), Universidad de San Andrés, Buenos Aires, Buenos Aires, Argentina; Faculty of Education, National University of Cuyo, Mendoza, Argentina; Global Brain Health Institute (GBHI), University of California San Francisco (UCSF); & Trinity College Dublin, Dublin, Ireland; Universidad de Santiago de Chile, Santiago, Santiago, Chile; BrainLat Institute, Santiago, Santiago, Chile; INECO Foundation, Buenos Aires, Argentina

## Abstract

**Background:**

Speech and language assessments are central to research on cognitive decline and Alzheimer's dementia (AD). Relevant measures support screening, diagnostic, monitoring, phenotyping, and prognostic procedures in a non‐invasive, automatable, cost‐efficient fashion. However, the field is marked by minimal linguistic diversity, with English‐speaking cohorts accounting for ≈70% of publications and most languages being underexplored or unexplored. Thus, we ignore whether proposed markers in the literature prove relevant to long‐neglected speech communities. Here we introduce results from a trans‐regional framework testing candidate speech markers of AD and cognitive decline in speakers of Kiswahili from Kenya, Spanish from Chile, and Portuguese from Brazil.

**Method:**

We used our multilingual Toolkit to Examine Lifelike Language (TELL) to record, preprocess, and analyze (semi)spontaneous speech from 600 participants, including early‐stage AD patients and healthy controls (HCs). We focused on speech timing metrics, given their relevance as potential markers of semantic memory retrieval effort across languages. First, by combining regression and correlation analyses, we examined whether such metrics predict clinical measures in Kiswahili, Spanish, and Portuguese speakers. Second, in a machine learning study, we examined whether speech timing markers established for English‐speaking patients generalize onto a Spanish‐speaking cohort.

**Results:**

Speech timing metrics predicted cognitive status performance in HCs and AD patients from Kiswahili‐speaking (CERAD‐DR: *r* = .73, *p* < .001) and Spanish‐speaking (ACE‐III: *r* = .63, *p* < .001) cohorts, as well as self‐rated reading abilities in Portuguese‐speaking AD patients (*r* = ‐.52, *p* = .03). Also, machine learning models trained on English‐speaking AD patients and HCs generalized best onto Spanish speakers when fed with speech timing than with word‐level features, both for identifying patients (AUC = .75) and for predicting their MMSE scores (*r* = .39, *p* < .001).

**Conclusion:**

This evidence underscores the utility of digital speech timing metrics to capture early markers of AD and cognitive decline across diverse speech communities. Notably, these differ not only in their primary language, but also in multiple socio‐cultural terms, emphasizing the approach's potential for trans‐regional dementia research. Further work in this direction, including ongoing harmonization efforts, will be central for pursuing equity in brain health research.

